# Supplementary material for: Bayesian refinement of protein structures and ensembles against SAXS data using molecular dynamics
Source: PLoS Comput Biol. 2017 Oct 18;13(10):e1005800. doi: 10.1371/journal.pcbi.1005800 (PMC5662244; doi:10.1371/journal.pcbi.1005800)
Supplement: S1 Archive — (BZ2) [file pcbi.1005800.s011.tar.bz2 › gromacs_4.62_bayesian_saxs_refi/share/html/online/g_clustsize.html]

g\_clustsize


| g\_clustsize Main Table of Contents |

**VERSION 4.6  
Sat 19 Jan 2013**

---

### Description

This program computes the size distributions of molecular/atomic clusters in
the gas phase. The output is given in the form of an .xpm file.
The total number of clusters is written to an .xvg file.

When the -mol option is given clusters will be made out of
molecules rather than atoms, which allows clustering of large molecules.
In this case an index file would still contain atom numbers
or your calculation will die with a SEGV.

When velocities are present in your trajectory, the temperature of
the largest cluster will be printed in a separate .xvg file assuming
that the particles are free to move. If you are using constraints,
please correct the temperature. For instance water simulated with SHAKE
or SETTLE will yield a temperature that is 1.5 times too low. You can
compensate for this with the -ndf option. Remember to take the removal
of center of mass motion into account.

The -mc option will produce an index file containing the
atom numbers of the largest cluster.

### Files

| option | filename | type | description |
| --- | --- | --- | --- |
| **-f** | traj.xtc | Input | Trajectory: xtc trr trj gro g96 pdb cpt |
| **-s** | topol.tpr | Input, Opt. | Portable xdr run input file |
| **-n** | index.ndx | Input, Opt. | Index file |
| **-o** | csize.xpm | Output | X PixMap compatible matrix file |
| **-ow** | csizew.xpm | Output | X PixMap compatible matrix file |
| **-nc** | nclust.xvg | Output | xvgr/xmgr file |
| **-mc** | maxclust.xvg | Output | xvgr/xmgr file |
| **-ac** | avclust.xvg | Output | xvgr/xmgr file |
| **-hc** | histo-clust.xvg | Output | xvgr/xmgr file |
| **-temp** | temp.xvg | Output, Opt. | xvgr/xmgr file |
| **-mcn** | maxclust.ndx | Output, Opt. | Index file |

### Other options

| option | type | default | description |
| --- | --- | --- | --- |
| **-[no]h** | bool | no | Print help info and quit || **-[no]version** | bool | no | Print version info and quit || **-nice** | int | 19 | Set the nicelevel || **-b** | time | 0 | First frame (ps) to read from trajectory || **-e** | time | 0 | Last frame (ps) to read from trajectory || **-dt** | time | 0 | Only use frame when t MOD dt = first time (ps) || **-tu** | enum | ps | Time unit: fs, ps, ns, us, ms or s || **-[no]w** | bool | no | View output .xvg, .xpm, .eps and .pdb files || **-xvg** | enum | xmgrace | xvg plot formatting: xmgrace, xmgr or none || **-cut** | real | 0.35 | Largest distance (nm) to be considered in a cluster || **-[no]mol** | bool | no | Cluster molecules rather than atoms (needs .tpr file) || **-[no]pbc** | bool | yes | Use periodic boundary conditions || **-nskip** | int | 0 | Number of frames to skip between writing || **-nlevels** | int | 20 | Number of levels of grey in .xpm output || **-ndf** | int | -1 | Number of degrees of freedom of the entire system for temperature calculation. If not set, the number of atoms times three is used. || **-rgblo** | vector | 1 1 0 | RGB values for the color of the lowest occupied cluster size || **-rgbhi** | vector | 0 0 1 | RGB values for the color of the highest occupied cluster size |

---

http://www.gromacs.org  
gromacs@gromacs.org
